# Genome-wide single nucleotide polymorphism (SNP) data reveal potential candidate genes for litter traits in a Yorkshire pig population

**DOI:** 10.5194/aab-66-357-2023

**Published:** 2023-11-23

**Authors:** Yu Zhang, Jinhua Lai, Xiaoyi Wang, Mingli Li, Yanlin Zhang, Chunlv Ji, Qiang Chen, Shaoxiong Lu

**Affiliations:** 1 Faculty of Animal Science and Technology, Yunnan Agricultural University, Kunming, 650201, China; 2 Yunnan Fuyuefa Livestock and Poultry Feeding Company Limited, Kunming, 650300, China

## Abstract

The litter trait is one of the most important economic traits, and increasing litter size is of great economic value in the pig industry. However, the molecular mechanisms underlying pig litter traits remain elusive. To identify molecular markers and candidate genes for pig litter traits, a genome-wide association study (GWAS) and selection signature analysis were conducted in a Yorkshire pig population. A total of 518 producing sows were genotyped with Illumina Porcine SNP 50 BeadChip, and 1969 farrowing records for the total number born (TNB), the number born alive (NBA), piglets born dead (PBD), and litter weight born alive (LWB) were collected. Then, a GWAS was performed for the four litter traits using a repeatability model. Based on the estimated breeding values (EBVs) of TNB, 15 high- and 15 low-prolificacy individuals were selected from the 518 sows to implement selection signature analysis. Subsequently, the selection signatures affecting the litter traits of sows were detected by using two methods including the fixation index (FST) and θπ . Combining the results of the GWAS and selection signature analysis, 20 promising candidate genes (*NKAIN2*, *IGF1R*, *KISS1R*, *TYRO3*, *SPINT1*, *ADGRF5*, *APC2*, *PTBP1*, *CLCN3*, *CBR4*, *HPF1*, *FAM174A*, *SCP2*, *CLIC1*, *ZFYVE9*, *SPATA33*, *KIF5C*, *EPC2*, *GABRA2*, and *GABRA4*) were identified. These findings provide novel insights into the genetic basis of pig litter traits and will be helpful for improving the reproductive performances of sows in pig breeding.

## 1 Introduction

Litter traits, mainly including the total number born (TNB), the number born alive (NBA), piglets born dead (PBD), and litter weight born alive (LWB) (Wu et al., 2018), are some of the most important economic traits in the pig industry. Improving litter size is a primary objective and has been a central focus in breeding programs for several decades, particularly in well-organized breeding systems like the Canadian Centre for Swine Improvement (http://www.ccsi.ca/, last access: 20 October 2022) (Zhang et al., 2019). However, due to the low heritability of these traits and the cumulative small effects of multiple genes, genetic improvement for these reproductive traits is extremely limited by traditional breeding techniques based on the best linear unbiased predictions (BLUPs) (Holm et al., 2004). Therefore, it is necessary to further unravel the underlying genetic architecture of these traits to improve the breeding schemes. With the development of high-throughput genotyping technology and the improvement in genetic statistical methods, some methods have been widely used in the genetic dissection of important economic traits in livestock, such as a genome-wide association study (GWAS) and selective sweep analysis.

Since the publication of domestic pig genome data in 2005 (Wernersson et al., 2005), GWASs have been widely used to identify molecular markers, genes, and quantitative trait loci (QTL) for reproductive traits in pigs, such as litter size (Uzzaman et al., 2018; Jiang et al., 2020; Chen et al., 2019), the number of teats (Bovo et al., 2021; Moscatelli et al., 2020), ovulation rate (Schneider et al., 2014; He et al., 2017), and gestation length (Hidalgo et al., 2016; See et al., 2019). In addition, phenotypic variability offers the opportunity to investigate the molecular basis of differentiation between populations (D'Alessandro et al., 2020). In the past decade, selection signature has been used to identify candidate regions of chromosomes of phenotypic traits in pigs. For instance, Li et al. (2020) identified 1017 selective windows containing 280 genes by comparing genomes of Large White pigs with high and low prolificacy. Wu et al. (2020) detected 20 candidate genes including *PALM2-AKAP2*, *NANS*, *TRAF7*, and *PACSIN1* for body size through the runs of homozygosity (ROH) islands and integrated haplotype homozygosity score (iHS) methods in Diannan small-ear pigs. To date, although a total of 986 QTLs associated with reproductive traits have been detected (https://www.animalgenome.org/cgi-bin/QTLdb/SS/, last access: 28 October 2022), a limited number of genes were reported for each reproductive trait, which could explain only a small proportion of genetic variance (Manolio et al., 2009). Hence, more molecular markers and genes need to be further explored.

The aim of the present study was to detect potentially important genetic variants and genes associated with four litter traits in the Yorkshire pig population by performing a GWAS and selective sweep analysis.

## 2 Materials and methods

### 2.1 Animals, phenotypic collection, and genetic parameter and estimated breeding value estimation

The pure-bred Yorkshire sows used in this study were raised under the same nutritional and feeding management at Fuyuefa Pig Breeding Farm (Kunming, China). Four litter traits – TNB, NBA, PBD, and LWB – were recorded for all sows. A total of 1969 litter records of 518 sows from parities 1 to 10 were collected during the year 2013 to 2020. Estimated breeding values (EBVs) for the four litter traits were calculated with the method of BLUP by tracing back four generations of pedigree information. The following animal repeatability model based on DMU software (http://dmiu.agrsci.dk, last access: 2 October 2022) was used to estimate the variance components and EBVs:

1 Y=Xfix+Zadd+Wsire+Pper+e,

where Y was the vector of observation (including TNB, NBA, PBD, and LWB), fix was the vector of fixed effects (year–season–parity), add was the vector of additive genetic effects, sire was the random effects with breeding boars, per was the vector of individual permanent environmental effects, e was a vector of residuals, and X , Z , and P were incidence matrices associated with fix , add , and per , respectively. The farrowing parities were classified into four levels (parities 1, 2, 3, and more than 3) in order to reduce the imbalance among years and parities.

Based on the estimated variance components for each trait and their standard errors (SEs) by employing the average information restricted maximum likelihood (AI-REML) algorithm of DMU software, heritabilities ( $h^{2}$ ) and genetic correlations ( $r_{A}$ ) and their SEs were calculated. Among them, $h^{2}$ and $r_{A}$ were calculated according to the formulas $h^{2}=\sigma_{a}^{2}/(\sigma_{a}^{2}+\sigma_{s}^{2}+\sigma_{\mathrm{pe}}^{2}+\sigma_{e}^{2})$ and $r_{A}=\mathrm{cov}(a_{1}a_{2})/\sigma_{a_{1}}\sigma_{a_{2}}$ , where $\sigma_{a}^{2}$ , $\sigma_{s}^{2}$ , $\sigma_{\mathrm{pe}}^{2}$ , and $\sigma_{e}^{2}$ were sequentially additive genetic variance, sire variance, permanent environmental variance, and residual variance of the same trait, and $\mathrm{cov}(a_{1},a_{2})$ , $\sigma_{a_{1}}$ and $\sigma_{a_{2}}$ were the genetic covariance of two traits and genetic deviations of trait 1 and trait 2.

The EBVs of 518 sows for TNB were sorted from the lowest to the highest, and the highest and the lowest 15 individuals, namely high-prolificacy (HP) and low-prolificacy (LP) groups, were selected for subsequent selection signature analysis. The differences in the phenotypic values and EBVs between HP and LP groups were tested by the procedure GLM and TTEST in SAS9.2 (SAS Institute, Inc., Cary, North Carolina), respectively. The statistical model was described as follows:

2yijkl=μ+Groupi+Parityj+Yeark+Seasonl+eijklm,3yij=μ+Groupi+eij.

For phenotypic values, significance tests were performed using model 2, where $y_{ijkl}$ was the observed value of the trait, μ was the mean of the population, Parity ${}_{j}$ was an effect of parity, Year ${}_{k}$ was an effect of the farrowing year, Season ${}_{l}$ was an effect of the farrowing season, and $e_{ijklm}$ was an effect of residuals. For EBVs, a significance test was performed using model 3, where $y_{ij}$ was the EBV of individual; μ and $e_{ij}$ were the mean of EBV and residual effects, respectively; and Group ${}_{i}$ was the grouping effect of HP and LP.

### 2.2 Genomic DNA extraction and genotyping

Totals of 518 individuals were genotyped and used for the GWAS analysis, of which 30 individuals were used for selection signature analysis. Genomic DNA was extracted from ear tissue following the manufacturer's instructions using the FinePure Universal Genomic DNA Kit. Then, DNA quality was detected using agarose gel electrophoresis and NanoDrop 2000 (Thermo Scientific, Waltham, MA, USA). Samples with clear and bright bands (no stray bands) and an A260 / 280 ratio between 1.7 and 2.1 were retained. Genotyping was performed using the Illumina Porcine SNP 50 BeadChip, which covered a comprehensive set of 51 315 single nucleotide polymorphisms (SNPs) distributed across the entire porcine genome. The genotyping was carried out by Beijing Compass Biotechnology Co., Ltd., located in Beijing, China.

### 2.3 Data quality control

For phenotypic data, descriptive statistics of the four litter traits were performed using the proc MEANS in SAS9.2 (SAS Institute, Inc., Cary, North Carolina) and tested for normality using the Shapiro.test function. For genotypic data, quality control was performed using the PLINK v1.9 software (Purcell et al., 2007). The filter criteria were as follows: (1) SNP call rate <0.90 ; (2) minor allele frequency <0.01 ; and (3) animals with a call rate <0.95 ; (4) $P<1\times 10^{-6}$ for a Hardy–Weinberg equilibrium (HWE) test. Moreover, SNPs without positional information or those located on the sex chromosome were also removed from the dataset. Missing genotypes were imputed using Beagle5.2 (Browning et al., 2018).

### 2.4 Genome-wide association study

To control the false positive results caused by population stratification, gcta software was used to analyze the population structure. All quality-controlled SNPs were pruned using the indep-pairwise parameter in the PLINK v1.9 software (Purcell et al., 2007), and 10 201 independent SNPs were generated using a window size of 25 SNPs, a step size of 5 SNPs, and an $r^{2}$ threshold of 0.4 (Gu et al., 2011). These 10 201 independent SNPs were then used for population stratification correction.

The GWAS analysis was implemented using a repeatability model on the basis of the GMAT software (Ning et al., 2019) for four traits. The statistical model was described as follows:

4 Y=Xβ+Zkγk+ξ+p+e,

where Y was the phenotype vector (including TNB, NBA, PBD, and LWB); Xβ was the fixed effect, including the population structure effect of sow farrowing year, season, and parity; Zkγk was the marker effect to be tested; $\xi ∼N(0,K\varphi^{2})$ was the polygene effect; $p∼N(0,I\sigma p^{2})$ was the system environment effect; and $e∼N(0,I\sigma^{2})$ was the residual effect. K in polygenic effects was the relatedness matrix inferred from markers. To avoid overcorrection, we used the previously calculated independent SNPs to determine the significance threshold; the threshold P value for genome-wide significant association was $4.90\times 10^{-6}$ (0.05/10 201), and for suggestive association, it was $9.80\times 10^{-5}$ (1/10 201) (Gu et al., 2011). Then, the genomic inflation factor λ was calculated using R.

### 2.5 Selective sweep analysis

To further illustrate the genetic differentiation between HP and LP sows, the following methods were performed. (1) Maximum likelihood (ML) trees were constructed using the MEGA7.0 software (Kumar et al., 2016) based on 41 314 genetic variants in each group and visualized using the ggtree package of R (Yu et al., 2018). (2) Principal component analysis (PCA) was performed using the PLINK v1.9 software (Purcell et al., 2007). The results were visualized using the ggplot2 package of R.

Subsequently, the selection signatures across genomes of HP and LP sows were detected using two statistics: the fixation index (FST) and the polymorphism level ( θπ ratio). FST and θπ ratio were implemented using a 100 kb sliding window with a step size of 10 kb based on the vcftools software (Danecek et al., 2011). FST was the inbreeding coefficient of a subpopulation and was used for estimating the degree of pairwise genomic differentiation on candidate genes between pairs of subpopulations, and π was the expected heterozygosity per site derived from the average number of sequence differences in a group of samples (Lu et al., 2019). The top 5 % of regions were selected as candidate regions for selection by two methods. To make the results more reliable, the overlapping regions from the two methods were selected as key candidate regions.

### 2.6 Identification of candidate genes

For the GWAS, the potentially significant SNPs were annotated to the Sus Scrofa 10.2 reference genome assembly using the UCSC genome browser (Xu et al., 2019). Genes containing potentially significant SNPs or situated near the identified potentially significant SNPs (i.e., less than 300 kb away from potentially significant SNPs) were selected. For selective sweep analysis, genes located within the selective regions were regarded as candidate genes. Then, functional enrichment analysis of the gene ontology (GO) and Kyoto Encyclopedia of Genes and Genomes (KEGG) pathways was performed for the candidate genes by using the Database for Annotation, Visualization, and Integrated Discovery (DAVID) (Sherman et al., 2022).

## 3 Results

### 3.1 Descriptive statistics of phenotypes

The results of a normality test showed that all the data of the four traits followed a normal distribution ( P>0.88 ). The descriptive statistics of phenotypic values and EBV information for the four litter traits in this study are shown in Table 1. The phenotypic mean values of TNB, NBA, PBD, and LWB were 11.37, 9.67, 1.71, and 13.89, and the mean EBVs of the four traits were -0.09 , -0.08 , 0.01, and -0.68 , respectively. There were large phenotypic variations in the four traits within the population. As shown in Table 2, the heritabilities of TNB, NBA, PBD, and LWB were 0.07, 0.06, 0.02, and 0.05, respectively. Strong positive genetic correlations were observed among the three traits, and the genetic correlation between TNB and NBA was 0.88.

**Table 1:** Phenotypic values and EBVs of four litter traits.

Trait ${}^{a}$	Phenotypic value ± SD	EBV ± SD
	( $n=1969^{b}$ )	( $n=518^{c}$ )
TNB	11.37 ± 2.25	-0.09 ± 10.38
NBA	9.67 ± 2.30	-0.08 ± 14.78
PBD	1.71 ± 0.31	0.01 ± 9.07
LWB (kg)	13.89 ± 4.00	-0.68 ± 28.17

${}^{a}$ TNB: total number born; NBA: number born alive; PBD: piglets born dead; LWB: litter weight born alive. ${}^{b}$ n : number of litters. ${}^{c}$ n : number of sows.

**Table 2:** Trait heritabilities and genetic correlations among four litter traits.

Trait ${}^{*}$	TNB	NBA	PBD	LWB
TNB	0.07 (0.01)			
NBA	0.88 (0.03)	0.06 (0.01)		
PBD	-0.05 (0.08)	-0.28 (0.12)	0.02 (0.01)	
LWB	0.62 (0.06)	0.84 (0.03)	-0.16 (0.09)	0.05 (0.01)

${}^{*}$ The data on the diagonal are heritability (standard error), and data below the diagonal are genetic correlation (standard error) between two traits.

The phenotypic values and EBVs for the four litter traits of HP and LP groups were shown in Table 3. For TNB, the mean phenotypic value and EBV of the HP group were 14.85 and 19.91, which is significantly higher than for the LP group of 10.27 and -20.34 ( P<0.001 ). The significance differences of NBA and LWB between the two groups were all consistent with those of TNB, but there were no significant differences ( P>0.05 ) in PBD between HP and LP sows.

**Table 3:** The phenotypic values and EBVs for litter traits of HP and LP sows.

Group ${}^{*}$	n	TNB		NBA		PBD		LWB (kg)	
		Phenotypic	EBV ± SD	Phenotypic	EBV ± SD	Phenotypic	EBV ± SD	Phenotypic	EBV ± SD
		value ± SD		value ± SD		value ± SD		value ± SD	
HP	15	14.85 ± 0.49 ${}^{A}$	19.91 ± 1.13 ${}^{A}$	12.08 ± 0.53 ${}^{A}$	19.95 ± 2.31 ${}^{A}$	1.67 ± 0.06	1.51 ± 7.18	17.66 ± 0.77 ${}^{A}$	28.01 ± 3.68 ${}^{A}$
LP	15	10.27 ± 0.34 ${}^{B}$	-20.34 ± 1.14 ${}^{B}$	8.25 ± 0.37 ${}^{B}$	-22.58 ± 2.30 ${}^{B}$	1.73 ± 0.08	1.76 ± 6.91	12.45 ± 0.54 ${}^{B}$	-32.21 ± 3.61 ${}^{B}$

${}^{*}$ Different capital letters (A, B) indicate significance with P<0.001 .

### 3.2 SNP identification

Through quality control, 296, 177, and 8890 SNPs were excluded from the raw genotype dataset due to missing genotype data, HWE test, and minor allele frequency (MAF), respectively (Fig. S1 in the Supplement). For selective sweep analysis, a total of 30 individuals and 41 314 SNPs remained for further analysis. For the GWAS analysis, 41 314 SNPs from 518 individuals were retained for subsequent analysis.

### 3.3 Genome-wide association study

**Figure 1:**
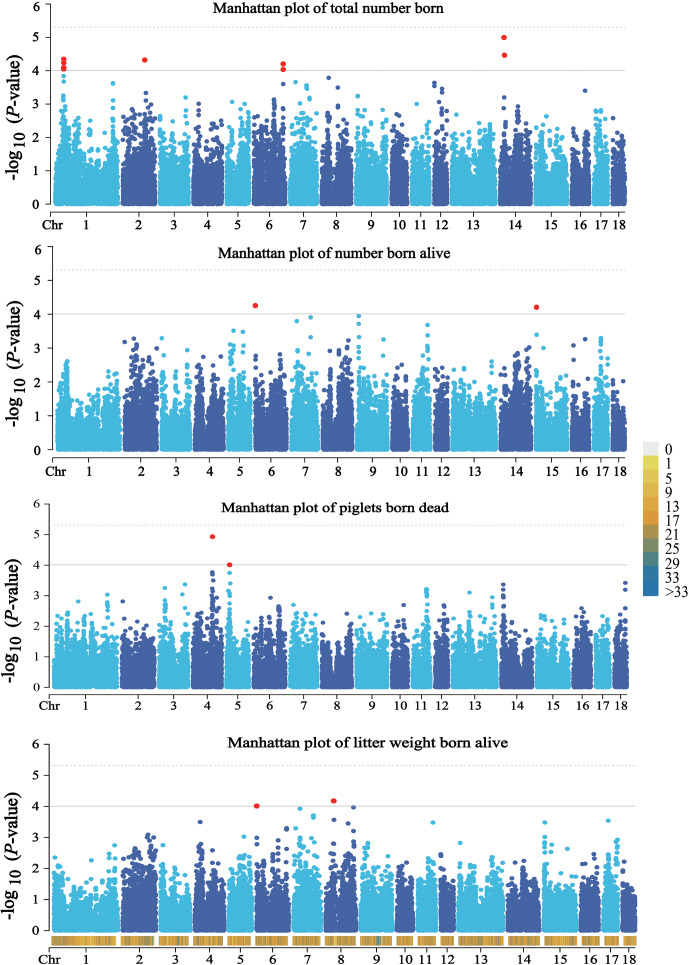
Manhattan plots of the observed P values for four litter traits. The black horizontal lines indicate the suggestive significance ( $9.80\times 10^{-5}$ ) thresholds, and the horizontal dashed lines indicate the genome-wide significance ( $4.90\times 10^{-6}$ ) threshold. The red dots stand for the potential SNPs associated with the traits of TNB, NBA, PBD, and LWB. The different colors from top to bottom in the color scale stand for the number of SNPs on the chromosomes.

A total of 14 SNPs that reached the suggestive significance level were detected to have an association with one of the tested litter traits (Table 4) and were defined as the potential SNPs for TNB, NBA, PBD, and LWB. And the λ values were 1.09, 1.09, 1.06, and 1.05 for the four traits, respectively. The results of population structure analysis were shown in Fig. S2. The manhattan plots and Q–Q plots across the whole genome for the four traits were shown in Figs. 1 and S3. By extending 300 kb downstream and upstream of the potential SNPs, a total of 49 different functional genes were discovered. Combining GO analysis, KEGG analysis, and the published literature, 13 functional genes were identified as promising candidates for litter traits (Table 4).

For TNB, we identified nine SNPs corresponding to eight related genes. The most significant SNP was rs80979178, located in the downstream region of the *CLCN3* gene on chromosome 14. The other two SNPs (rs81393707 and rs81393705) were located in the introns of the *CLIC1* and *ZFYVE9* genes on chromosome 6. In addition, the remaining six SNPs were located on chromosomes 1, 2, 6, and 14, respectively. For NBA and LWB, a total of three SNPs corresponding to five genes were identified. The rs81478807 was located in the introns of the *KIF5C* gene on chromosome 15. Besides, *GABRA2* and *GABRA4* were found in the upstream region of rs81293800 located on chromosome 8. Interestingly, the rs81330557 located in the upstream region of the *SPATA33* gene on chromosome 6 was significantly associated with NBA and LWB. For PBD, we identified two SNPs, which were located on chromosomes 4 and 5. Furthermore, GO and KEGG results showed that these genes were significantly enriched in some biological processes (BPs) and pathways related to solute transport, cell differentiation, embryonic development, and central regulation of reproduction. The functional genes of TNB were enriched in solute transport, and cell differentiation, such as *CLCN3* and *CLIC1*, was enriched in chloride transport (GO:0006821) and chloride transmembrane transport (GO:1902476). Meanwhile, *CLIC1* was enriched in the glutathione metabolic process (GO:0006749). Additionally, *SCP2* and *CBR4* were enriched in the fatty acid metabolism signal pathway (ssc01212). For LWB, the functional genes were involved in the central regulation process of reproduction; for example, *GABRA2* and *GABRA4* were enriched in the regulation of postsynaptic membrane potential (GO:0060078), synaptic transmission, and the GABAergic (GO:0051932) and gamma-aminobutyric acid signaling pathway (GO:0007214) (Table S1 in the Supplement).

**Table 4:** The potential significant SNPs and candidate genes associated with the four litter traits. SSC: *Sus scrofa* chromosome.

Trait	SNP	SSC	Position (bp)	P value	Effect	MAF ${}^{a}$	Candidate gene ${}^{b}$
TNB	rs80979178	14	21 826 839	$1.0\times 10^{-5}$	0.409	0.281	*CLCN3*, *CBR4*
	rs332515193	14	19 190 881	$1.3\times 10^{-5}$	0.398	0.475	*HPF1*
	rs80946136	1	42 826 453	$4.5\times 10^{-5}$	0.358	0.464	/
	rs80940699	2	110 388 942	$4.8\times 10^{-5}$	0.492	0.154	*FAM174A*
	rs80811774	1	42 802 898	$5.8\times 10^{-5}$	0.355	0.466	/
	rs81393707	6	147 556 506	$6.2\times 10^{-5}$	0.344	0.352	*SCP2*, *CLIC1*
	rs338264465	1	42 528 991	$8.1\times 10^{-5}$	0.345	0.456	*NKAIN2*
	rs325033187	1	42 656 427	$8.9\times 10^{-5}$	0.359	0.347	*NKAIN2*
	rs81393705	6	147 582 955	$9.2\times 10^{-5}$	0.336	0.349	*ZFYVE9*
NBA	rs81330557	6	469 860	$5.6\times 10^{-5}$	0.575	0.096	*SPATA33*
	rs81478807	15	2 788 023	$6.2\times 10^{-5}$	0.370	0.224	*KIF5C*, *EPC2*
PBD	rs80883215	4	97 489 219	$1.1\times 10^{-5}$	0.275	0.482	/
	rs81382621	5	15 161 118	$9.7\times 10^{-5}$	0.301	0.232	/
LWB	rs81293800	8	38 814 862	$6.7\times 10^{-5}$	-0.904	0.079	*GABRA2*, *GABRA4*
	rs81330557	6	469 860	$9.7\times 10^{-5}$	0.828	0.096	*SPATA33*

${}^{a}$ MAF: minor allele frequency. ${}^{b}$ Candidate gene: the SNP-containing or nearest annotated genes for each potential SNP.

### 3.4 Selective sweep analysis

**Figure 2:**
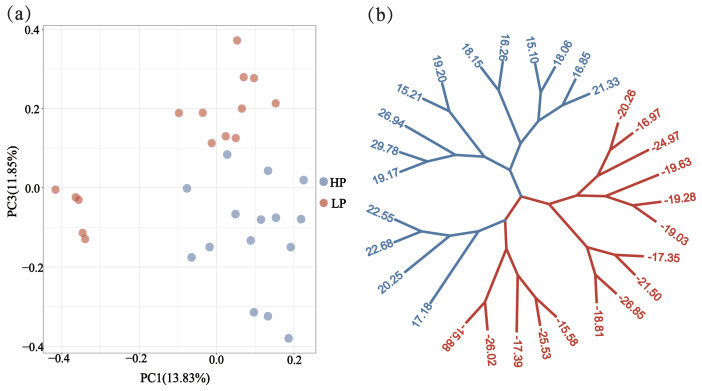
Population structure and relationship between the HP and LP sows. **(a)** PCA clustering. **(b)** Maximum likelihood (ML) trees of the relationship between the HP and LP sows. Light blue and pink branches indicate sows in the HP and LP group, respectively, and the value of each leaf node represents the individual EBV of TNB.

**Figure 3:**
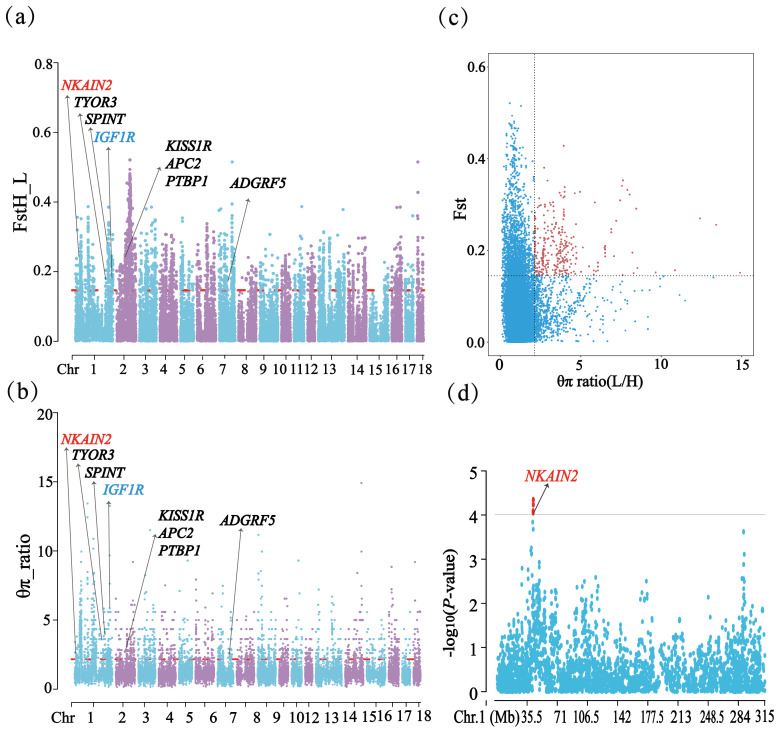
FST, θπ ratio, and the GWAS for TNB on chromosome 1 related results in HP and LP sows. **(a)** Distribution of FST values among autosome chromosomes. **(b)** Distribution of θπ ratio values among autosome chromosomes. **(c)** The final selection regions based on two statistics. **(d)** Distribution of the GWAS for TNB on chromosome 1.

Through phylogenetic tree analysis and PCA, significant genetic differentiation was detected between the HP and LP sows (Fig. 2). For the two groups, the PC1 and PC3 explained 13.83 % and 11.85 % of the total variations, respectively. Similar to PCA clustering analysis, ML trees also revealed clear population structures. Selective sweep analysis based on FST and θπ ratio showed many small selective regions. For θπ ratio, the average nucleotide diversity was $7.52\times 10^{-6}$ and $7.71\times 10^{-6}$ for HP and LP sows, respectively. And the 10 019 windows (top 5 %) were selected (Table S2). Meanwhile, the 10 157 selected regions were identified for FST (Table S3). Then, the overlapped regions corresponding to the two approaches (threshold, 5 %; FST, 0.15; π ratio, 2.13) were defined as the key selection signatures, which contained 256 genes (Table S4). Furthermore, functional enrichment analysis revealed that 32 genes were significantly enriched in seven GO BPs and one KEGG pathway (Table S5), including proteolysis (GO:0006508), the regulation of cell differentiation (GO:0045595), the positive regulation of phospholipid biosynthetic process (GO:0071073), and neuropeptide signaling pathway (GO:0007218) and so on. One KEGG pathway (FoxO signaling pathway) was identified as having an association with follicular development and ovulation. Combined with the published results, eight genes (*NKAIN2*, *IGF1R*, *KISS1R*, *TYRO3*, *SPINT1*,* ADGRF5*, *APC2*, and *PTBP1*) were identified as potential candidate genes (Fig. 3).

## 4 Discussion

Litter traits such as TNB, NBA, PBD, and LWB are directly related to sow reproductive performance and affected pig production efficiency and economic profit. Therefore, it was extremely important to better understand the underlying genetic architecture of sow litter performance. In the present research, we used the Illumina Porcine SNP 50 BeadChip to genotype a Yorkshire pig population. Then, the GWAS based on the repeatability model and the selective sweep analysis were implemented.

For the GWAS, a total of 14 SNPs and 13 functional genes were significantly associated with litter traits, of which 12 genes were newly identified except for *ZFYVE9*.

The SNP rs81330557 on chromosome 6 was associated with NBA and LWB. This SNP was located within the upstream region of the spermatogenesis-associated 33 (*SPATA33*) gene, which is known as *C16orf55* and is conserved among mammalian species. Chen et al. (2013) showed that *SPATA33* was mainly expressed in spermatocytes, spermatogonia, and round spermatids and was related to spermatogenesis. Besides, it was reported that *SPATA33* interacted with sperm calcine phosphatase through the *PQIIIT* sequence, and *SPATA33* mutant mice displayed lower sperm motility due to the rigidity of the middle part of the sperm, impairing male fertility (Miyata et al., 2021).

The *CLCN3* encodes a member of the voltage-gated chloride channel (ClC) family, which plays a role in the acidification and transmitter transmission of GABAergic synaptic vesicles. The GO and KEGG results showed that *CLCN3* was significantly enriched in the BP of chloride transport. Besides, *CLCN3* had previously been reported to be the most likely candidate gene involved in the human sperm volume regulation of the Cl ${}^{-}$ channel and promoted endometriosis cell migration and invasion (Yeung et al., 2005; Guan et al., 2016).

Two SNPs (rs81393707 and rs81393705) on chromosome 6 were also associated with TNB and were located within the intron of chloride intracellular channel 1 (*CLIC1*) and a *FYVE*-type zinc finger containing nine (*ZFYVE9*) genes, respectively. *CLIC*, a member of the p64 family, was mainly found in the nucleus and had chloride channel activity in the nuclear and plasma membranes. This gene was a sensor of cellular oxidation, involved in reactive oxygen species (ROS) production and was highly expressed in human embryos (Fagerberg et al., 2014; Goodchild et al., 2009; Averaimo et al., 2010). *CLIC1* was enriched in two BP GO terms: chloride transport and glutathione metabolic process. Research has shown that the former might affect the stage-specific gradients of pH in the follicle cell, thereby regulating the intracellular environment, the development, and the function of follicles (Weiß and Bohrmann, 2019); the latter might be associated with pregnancy placental dysfunction, embryogenesis, and other processes (Beharier et al., 2021; Chen et al., 2022). There was report showed that *CLIC1* expression accompanied by a low accumulation of ROS could promote embryonic development (Cajas et al., 2020).

*ZFYVE9* also known as *SARA* was an *FYVE*-type zinc finger-containing protein (Di Guglielmo et al., 2003). Liu et al. (2013) showed that the knockout of *ZFYVE9* affected the early embryonic development of zebrafish. In pigs, *ZFYVE9* has been determined to be strongly associated with ovulation and the number of mummified foetuses by the GWAS (Onteru et al., 2012; Schneider et al., 2014). The results of association analysis by Li et al. (2020) also showed that *ZFYVE9* was significantly correlated with TNB, and our result was consistent with these results.

In addition, an additional four candidate genes identified by the GWAS (*HPF1*, *EPC2*, *GABRA2*, and *GABRA4*) were also found to be linked to litter traits. *HPF1* was a protein-coding gene that influenced chromatin binding activity and histone binding activity (Bilokapic et al., 2020). The decreased expression of *HPF1* affected the early development of zebrafish (Zhang et al., 2018). *EPC2* was mainly involved in the regulation of transcription by RNA polymerase II and has been reported to be associated with fertility in pigs (Chen et al., 2022). *GABRA2* and *GABRA4* are members of the *GABA-A* receptor gene family of heteromeric pentameric ligand-gated ion channels (Whiting et al., 1999) and significantly enriched in the BP of the regulation of postsynaptic membrane potential. Studies have shown that postsynaptic membrane potential regulation between neurons might affect the synthesis and release of reproductive hormones (DeFazio et al., 2019; Csillag et al., 2019). Therefore, the two genes might be related to reproductive hormone regulation and reproductive performance of pigs. It was speculated that the possible mechanism of the association of the two genes with reproductive performance regulated the secretion of estrogen by the regulation of brain development and transmission of neurotransmitters.

In the present study, only 14 potentially significant SNPs were detected by the GWAS. The main reason might be the limited size of the research population so that only SNPs or QTLs with large and moderate effects could be detected. Therefore, it is necessary to identify those SNPs or QTLs with small effects by expanding the sample size in future research.

Over the past 10 years, intensive breeding of the population has resulted in some genetic improvements in reproductive traits, especially in litter traits, which provided us with the opportunity to identify relevant candidate genes using selection signatures. According to earlier research, tests with extreme and moderate phenotypes in a single group could lessen the effects of the environment and the genetic differences among individuals (Jiang et al., 2016; Li et al., 2020). Therefore, to reveal the selective signatures of high prolificacy in Yorkshire pigs during domestication and breeding, we selected the region with the top 5 % FST and π ratio and identified 256 protein-coding genes. Thirty-two of these genes were significantly enriched in some BP GO terms; for example, *KISS1R* and *TYRO3* were enriched in neuropeptide signaling pathway, *SPINT1* was enriched in extracellular matrix organization, *ADGRF5* was enriched in a positive regulation of phospholipid biosynthetic process, and *APC2* and *PTBP1* were enriched in the regulation of cell differentiation, which indicated that the functional genes were mainly related to the signal peptide transduction, embryonic development, and reproductive process. Additionally, five genes including *IGF1R* were significantly enriched in the FoxO signaling pathway, which was an important pathway affecting follicular development and ovulation (Lin et al., 2021).

Combining the selective sweep analysis and published results, eight genes (*IGF1R*, *KISS1R*, *TYRO3*, *SPINT1*, *ADGRF5*, *APC2*, *PTBP1*, and *NKAIN2*) were identified as candidate genes for litter traits. *IGF1R* was a receptor for an insulin-like growth factor, which was involved in the regulation of cell growth and survival control after activation. Experiments in mouse models have shown that *IGF1R* deficiency or low expression in the endometrium led to abnormal implantation of mouse embryos, resulting in poor fertility (Kang et al., 2015; Zhou et al., 2021). In pigs, through selective sweep analysis, *IGF1R* was identified as the key gene for high-prolificacy traits in Meishan pigs (Zhao et al., 2018). Zhang et al. (2022) also identified *IGF1R* as a candidate gene related to pig reproductive traits in a selective sweep analysis of Wannan black pigs and commercial pigs. The present result was consistent with the results reported. Given this background, *IGF1R* might be a key gene responsible for phenotypic differences in reproductive traits in pigs. *CCND1* was a member of the highly conserved cyclin family. *KISS1R* was the receptor for the kisspeptin gene, and together they were involved in the neuroendocrine regulation of mammalian reproduction (Castano et al., 2009). A study also reported that *KISS1R* was able to promote oocyte maturation in vitro (Saadeldin et al., 2012). In pigs, it was reported that *KISS1R* might play a role in the physiological development of ovarian neovascularization (Basini et al., 2018). The *TYRO3* gene was part of a three-member transmembrane receptor kinase receptor family that regulates spermatogenesis and male fertility in mice (Chen et al., 2009). In addition, *TYRO3* modulated female reproduction by influencing gonadotropin-releasing hormone neuron survival and migration (Pierce et al., 2008). Recent studies have found that *SPINT2* was a functionally relevant placental protease inhibitor and that low circulating *SPINT1* was strongly associated with placental function defects (Kaitu'u-Lino et al., 2020; Murphy et al., 2021). It was inferred consequently that *SPINT1* might influence sow litter size and piglet birth weight by regulating placental function. Polypyrimidine bundle-binding protein 1 (*PTBP1*) is a member of the heterogeneous nuclear ribonucleoproteins (hnRNPs) subfamily. It is an important cellular regulator of messenger RNAs and plays an important role in early embryonic development (Suckale et al., 2011). *APC2* encodes a strongly conserved protein, which is preferentially expressed in post-mitotic neurons, and is involved in brain development through its regulation of neuronal migration and axon guidance (Almuriekhi et al., 2015). It was confirmed that *APC2* plays a key role in regulating ovarian Wnt signaling pathways and ovarian homeostasis (Mohamed et al., 2019). *ADGRF5*, also known as *GPR116*, has been demonstrated to be associated with pig litter size through the GWAS (Sell-Kubiak et al., 2022). Our study further demonstrated that *ADGRF5* is a potential candidate gene influencing litter traits in sows.

In addition, it is worth noting that the *NKAIN2* gene was detected by three methods (GWAS, FST, and θπ ratio), indicating that it was strongly selected in the present population and might be significantly associated with litter traits. It could be attributed to its crucial functions in neurotransmitter transmission, energy metabolism, and ion transport (Blokland et al., 2022; Rudkowska et al., 2015). *NKAIN2* encoding a transmembrane protein that interacts with the beta subunit of a sodium-/potassium-transporting ATPase may play essential roles in maintaining cell membrane potential, regulating neurotransmitter release, and ensuring proper ion homeostasis (Romania et al., 2013). These functions are vital for various reproductive processes in pigs. Neurotransmitters play a significant role in regulating reproductive functions, such as the modulation of the gonadotropin-releasing hormone (GnRH) and other hormones controlling the reproductive cycle. Previous studies have confirmed that germline alterations in the *TCBA1* gene were associated with developmental delay and typical physiological features (Yue et al., 2006). Therefore, we speculated that *NKAIN2*'s involvement in these processes might confer advantages in terms of fitness and reproductive success, leading to its strong selection in the population and potential association with litter traits. However, the function of this gene remains to be further explored.

## 5 Conclusions

In summary, a total of 20 promising candidate genes (*NKAIN2*, *IGF1R*, *KISS1R*, *TYRO3*, *SPINT1*, *ADGRF5*, *APC2*, *PTBP1*, *CLCN3*, *CBR4*, *HPF1*, *FAM174A*, *SCP2*, *CLIC1*, *ZFYVE9*, *SPATA33*, *KIF5C*, *EPC2*, *GABRA2*, and *GABRA4*) were identified as being associated with four litter traits (TNB, NBA, PBD, and LWB) in a Yorkshire pig population by using GWAS and selection signature methods. To the best of our knowledge, this study is the first to report that the *NKAIN2* gene is associated with pig litter traits by integrating a GWAS and selective sweep analysis. These findings provide novel insights into the genetic basis of pig litter traits, which will be helpful for implementing marker-assisted selection and genomic selection to improve reproductive performances in the pig industry.

## Supplement

10.5194/aab-66-357-2023-supplement The supplement related to this article is available online at: https://doi.org/10.5194/aab-66-357-2023-supplement.

## Data Availability

The datasets created and/or analyzed during the present study are not publicly accessible since the examined population is made up of the nucleus herd of the Yunnan Fuyuefa Livestock and Poultry Feeding Company Limited, but they are available from the corresponding author upon justifiable request.
