# Supplementary material for: Genome-wide single nucleotide polymorphism (SNP) data reveal potential candidate genes for litter traits in a Yorkshire pig population
Source: Arch Anim Breed. 2023 Nov 23;66(4):357–68. doi: 10.5194/aab-66-357-2023 (PMC10726026; doi:10.5194/aab-66-357-2023)
Supplement: The supplement related to this article is available online at: https://doi.org/10.5194/aab-66-357-2023-supplement. [file aab-66-357-supplement.zip › aab-66-357-2023-supplement-title-page.pdf]

Supplement of Arch. Anim. Breed., 66, 357–368, 2023
https://doi.org/10.5194/aab-66-357-2023-supplement
© Author(s) 2023. CC BY 4.0 License.

Open Access
Archives Animal Breeding

Supplement of

Genome-wide single nucleotide polymorphism (SNP) data reveal potential candidate genes for litter traits in a Yorkshire pig population

Yu Zhang et al.

Correspondence to: Qiang Chen (chq@sjtu.edu.cn) and Shaoxiong Lu (shxlu_ynau@163.com)

- aab-66-357-2023-supplement-title-page.pdf
- supplement
  - Fig S1.docx
  - Fig S2.docx
  - Fig S3.docx
  - Table S1.xlsx
  - Table S2.xlsx
  - Table S3.xlsx
  - Table S4.xlsx
  - Table S5.xlsx

The copyright of individual parts of the supplement might differ from the article licence.
