# Supplementary figures and images for: Genome-wide single nucleotide polymorphism (SNP) data reveal potential candidate genes for litter traits in a Yorkshire pig population
Source: Arch Anim Breed. 2023 Nov 23;66(4):357–68. doi: 10.5194/aab-66-357-2023 (PMC10726026; doi:10.5194/aab-66-357-2023)

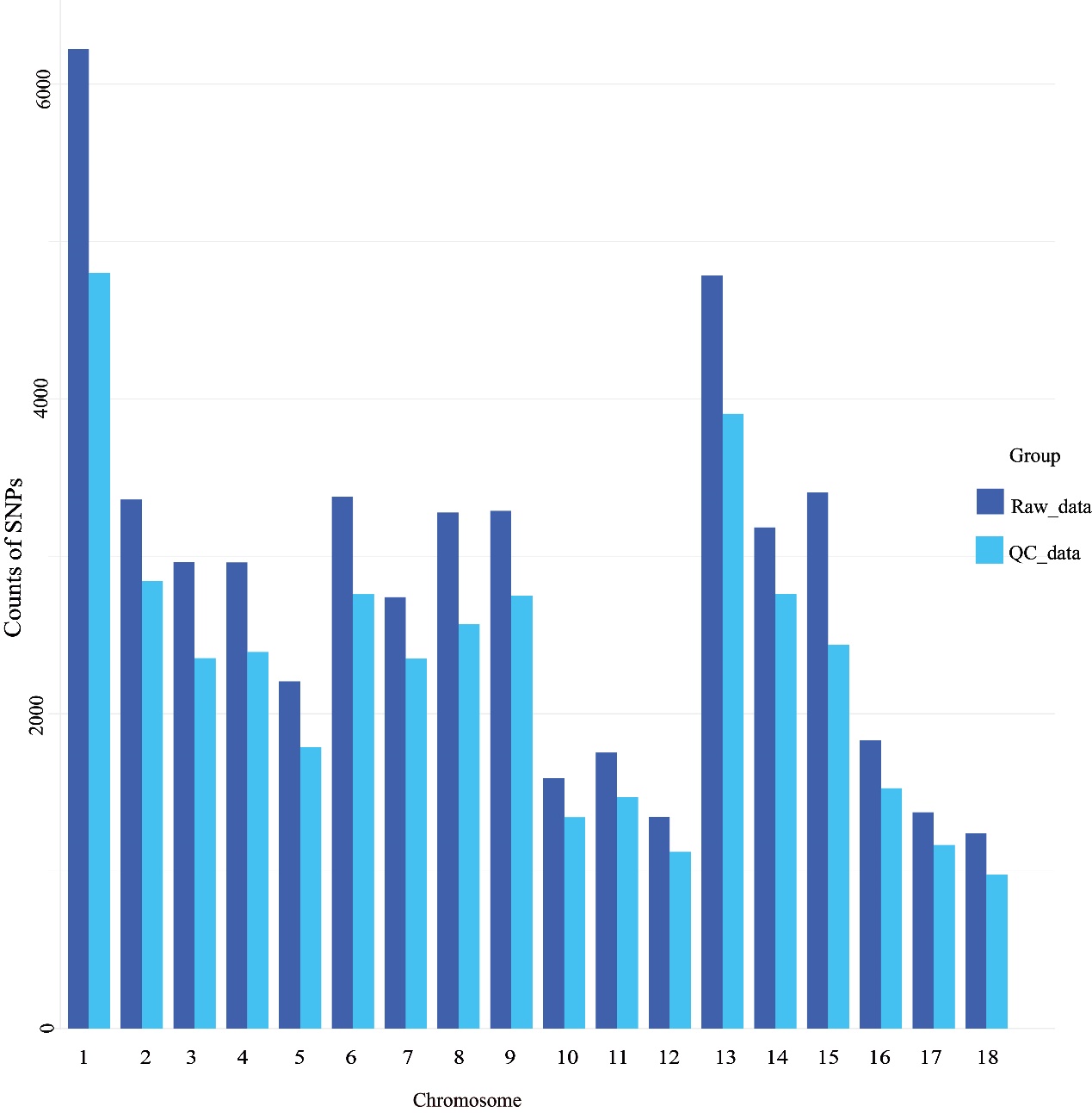


**Figure S1.** Distribution of autosomal SNPs before and after quality control.

Supplement: The supplement related to this article is available online at: https://doi.org/10.5194/aab-66-357-2023-supplement. [file aab-66-357-supplement.zip › supplement/Fig S1.docx]
